# Design of defect-chemical properties and device performance in memristive systems

**DOI:** 10.1126/sciadv.aaz9079

**Published:** 2020-05-08

**Authors:** M. Lübben, F. Cüppers, J. Mohr, M. von Witzleben, U. Breuer, R. Waser, C. Neumann, I. Valov

**Affiliations:** 1 Institut für Werkstoffe der Elektrotechnik II, RWTH Aachen University, Sommerfeldstraße 24, 52074 Aachen, Germany.; 2 JARA–Fundamentals for Future Information Technology, 52425 Jülich, Germany.; 3 Central Institute for Engineering, Electronics and Analytics, Forschungszentrum Jülich GmbH, 52425 Jülich, Germany.; 4 Peter-Grünberg-Institut (PGI 7), Forschungszentrum Jülich, Wilhelm-Johnen-Straße, 52425 Jülich, Germany.; 5 Heraeus Deutschland GmbH & Co. KG Heraeusstrasse 12-14, 63450 Hanau, Germany.

## Abstract

Future development of the modern nanoelectronics and its flagships internet of things, artificial intelligence, and neuromorphic computing is largely associated with memristive elements, offering a spectrum of inevitable functionalities, atomic level scalability, and low-power operation. However, their development is limited by significant variability and still phenomenologically orientated materials’ design strategy. Here, we highlight the vital importance of materials’ purity, demonstrating that even parts-per-million foreign elements substantially change performance. Appropriate choice of chemistry and amount of doping element selectively enhances the desired functionality. Dopant/impurity-dependent structure and charge/potential distribution in the space-charge layers and cell capacitance determine the device kinetics and functions. The relation between chemical composition/purity and switching/neuromorphic performance is experimentally evidenced, providing directions for a rational design of future memristive devices.

## Teaser

Impurities and dopants in memristive devices determine their switching kinetics, performance, and neuromorphic functionalities.

## INTRODUCTION

Memristive cells and devices are essential building units for future nanoelectronic architectures targeting alternative data processing paradigms such as cognitive/neuromorphic computing and alternative logic operations, all being milestones in assembles of global networks such as internet of things and creation of artificial intelligence. Memristive cells are energy efficient and scalable to almost atomic level, allowing read/write/erase operations within less than nanoseconds. These devices demonstrate multiple functionalities, such as nonvolatile memory and selector functions, ability to transit from digital to analog data storage and processing, sensor activities, etc. (*1*). They are extremely stable against high-energy particles and electromagnetic waves, thus preventing soft errors and can operate in a unprecedently broad temperature range from 4 K (*2*, *3*) to 600 K (*4*), making their application in space technologies and in general application under harsh conditions highly desirable.

In the recent years, the application of memristive devices evidenced remarkable progress in areas of downscaling, pattern/face recognition, integration in chips, arrays, and complex architectures (*1*, *5*–*9*). However, in most cases, the materials for devices are selected on the basis of empirical rules, depending on the particular operation type and/or the specific application. Furthermore, same materials are often used for different applications without identifying the reasons for the different performances. Thus, an essential fundamental aspect is still not addressed—the relation between the intrinsic material properties of the switching film and the device performance and functionalities.

All memristive functionalities (irrespective on the particular operation type and material system) rely on the change of fundamental material properties such as electric and/or magnetic resistances as a function of external stimuli. These stimuli can be different in nature, such as voltage, current, light, component, or absolute pressure and temperature, and must lead to measurable differences in at least one of these fundamental parameters. Mostly used are changes in the electronic conductivity (resistance), as observed in redox-based randon access memories (ReRAMs), phase change memories (PCM), magnetic random access memories (MRAMs), spin-transfer torque random access memories (STT-RAMs), Mott transition–based RRAMs, etc.

There is, however, an important point that is, up to date, overlooked—dielectric, electronic, and/or magnetic properties depend strongly on the concentration and chemistry of impurities in the material. These impurities must be considered in most cases as doping and may change not only the defect chemistry but also the capacitance, the electronic structure, the space-charge layers, and the resulting kinetic and thermodynamic properties. A prominent example in that respect is the development of the silicon-based industry in the 60s. It had begun only because of realizing the importance of extreme purity required for Si and Ge components, when Siemens researchers succeeded in purification of Si by zone melting (*10*) to reduce residual impurities. Doping strategies have been further developed (concentrations in parts-per-million range) to enhance one or other electronic property in these high-purity semiconductor materials. The issue of materials’ purity has not been considered yet for memristive devices, despite that changes in magnetic properties (*11*, *12*) and phase-change behavior (*13*, *14*) induced by foreign components have been reported.

This study highlights the relation between material purity, chemistry, and concentrations of intrinsic/extrinsic doping, and device performance and functionalities. We evaluate and demonstrate how concentration and chemistry of impurities modify the material properties and the structure and thickness of the space-charge layer upon doping SiO_2_ of highest purity with various elements. These factors determine the switching kinetics, device performance, and functionalities. The levels of doping that we use are below the impurity levels of materials typically used for memristive devices. Depending on the type and amount of the doping element, we can enhance or suppress particular characteristics such as fast/slow switching, short/long retention time, short-term plasticity (STP), or long-term plasticity (LTP), potentiation, etc. Our results apply to a broad spectrum of memristive devices and materials and provide general directions for selective device improvement.

## RESULTS

### Defect chemical properties of amorphous oxides

The physicochemical properties of amorphous oxide materials used for memristors are typically discussed in the context of point defects in crystalline solids such as oxygen vacancies, interstitial ions, etc. Although this approach is widely spread in memristive community and appears in many cases useful, there are several important arguments that limit this approximation. (i) Definition of point defects is strictly valid only for crystalline solids with a periodic lattice order. (ii) Point defect dynamics can be difficultly compared at low (e.g., room) and high temperatures. (iii) At lower temperatures, point defects are usually associated within the oxide matrix, forming dipoles (which have not yet been considered).

These dipoles and/or additional components change the permittivity of the main matrix. We used this effect to propose an effective way to estimate the defect-chemical concentration, by measuring the dielectric characteristics. This approach allows concluding on the impurities/dopant concentrations and relative bond strengths that, in turn, are closely related to the interatomic/ionic interactions and correlated device characteristics, allowing to predict the memristive properties of the nanoscale films.

We selected for the model study SiO_2_ as switching material in electrochemical metallization (ECM) device of the type Cu/SiO_2_/Pt. This system is extensively studied, and underlying electrochemical processes and switching kinetics are well documented and understood (*15*–*20*). Additional experiments with Ta/Ta_2_O_5_/Pt [valence change memory (VCM)] device have experimentally confirmed the theoretically expected general validity of the effects and conclusions, based on the common electrochemical fundamentals of all ReRAM systems (*21*).

The main reference material is pure SiO_2_ (purity degree, 8N+). To distinguish and highlight the effects of impurities/dopants, we doped the 8N SiO_2_ by copper (to provide mobile ions), aluminum, and gallium in different combinations. As the solubility of Cu in SiO_2_ is limited and it tends to form metallic clusters, Al^3+^ and Ga^3+^ ions are forming negatively charged cation point defects (substituting Si^4+^) and thus, acting as a counter charge of Cu^*x*+^, allowing that Cu ions to remain dissolved/unclustered in oxide matrix. Thus, Al and Ga doping increases (and controls/determines) the solubility of the Cu ions. Unlike other approaches using thermal diffusion (*22*) or photodiffusion (*23*), we used sputter targets with defined compositions, allowing us to have highly uniform concentration profiles throughout the switching layer (Supplementary text S1).

### Influence of impurities/doping and moisture on the defect chemical properties

Typical defects in SiO_2_ are protons and hydroxyl groups that can be weakly bonded (physisorption) and tightly (chemisorption) or directly incorporated in the Si-O network (*24*), oxygen-deficiency centers, dangling bonds, interstitial oxygen, and several more options [e.g., see (*25*)]. The impurities/doping are classified as volatile that are also mobile (e.g., moisture/protons) and nonvolatile (e.g., Al and Ga). Cu is considered as volatile as it can be extracted from or injected into the oxide (electrochemically) without being part of the original matrix and requiring additional charge compensation.

We have observed that dopants in SiO_2_ matrix (starting from parts-per-million range) significantly change its dielectric properties. Because of the different electron affinities of the foreign elements and/or defects, stronger or weaker interatomic electrostatic interactions are induced, leading to formation of dipoles, changing the permittivity and capacitance of the film, making it possible to detect even small amounts of foreign elements.

**Fig. 1:**
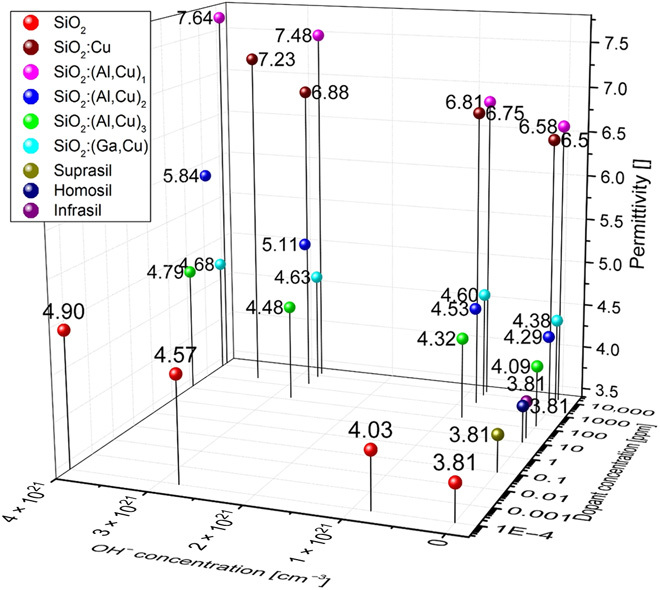
Permittivity values for pure and doped SiO_2_ as a function of OH^−^ concentration (water content) and dopant concentration. Highly pure SiO_2_ resembles water-free undoped Suprasil W. The permittivity values for the sputtered samples were measured in vacuum, at ~35% relative humidity (RH) and > 90% RH. We calculated the residual OH^−^ concentration in SiO_2_ films according to (*42*). This calculation procedure was also used for doped SiO_2_. For all materials, the permittivity is increasing with increasing OH^−^ (humidity) concentration, following linear relation $\varepsilon_{s}=3.8073+2.72*10^{-22}\frac{{\text{cm}}^{3}}{\text{ion}}*N$ (*42*), where *N* is the concentration of OH^−^defects per cubic meter. The increase in Al content leads to a much higher polarizability of the material, whereas Ga doping has lower impact on ε. The concentrations and permittivities of the glasses Suprasil, Homosil, and Infrasil are extracted from (*43*), (*44*), and (*42*), respectively. The method for determining dopant concentration by dielectric measurements has a sensitivity even in 1-ppm range (fig. S4).

In Fig. 1, pure and differently doped SiO_2_ films and their permittivity are plotted as a function of nonvolatile (Al and Ga) and volatile (Cu and moisture uptake in SiO_2_) doping concentrations. The permittivity of both pure and doped SiO_2_ films increases with increasing humidity levels following a linear relation (fig. S4), where for very high *p*_H2O_, the uptake of moisture begins to saturate. Similar results were obtained for Ta_2_O_5_ (fig. S4).

**Fig. 2:**
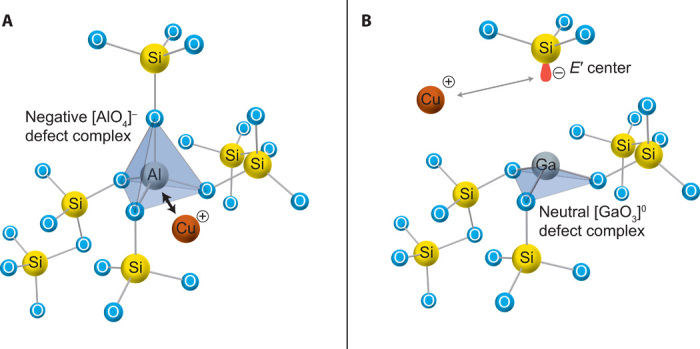
Schema of the atomistic configuration in SiO_2_ amorphous films around a substitutional atom. (**A**) Al ion replaces a Si ion. Because of the electron configuration of the Al, the local charge surplus can bind Cu^+^/Cu^2+^ ions in the vicinity of the Al atom. (**B**) Ga substitution. Ga is forming on [GaO_3_]^0^ complex, and Cu ions interact only with the *E*′ center of the [SiO_3_]^−^. This bond is weaker compared to that between Cu and [AlO_4_]^−^, resulting in a less pronounced polarization and smaller influence on the permittivity of the SiO_2_ matrix as function of the concentration.

Extrinsic dopants, e.g., Al or Ga, additionally increase the dielectric constant. The formation of dipoles between Al ion (relative charge in Kroeger-Vink notation $Al_{\text{Si}}^{\prime }$) that can be considered an acceptor and Cu ion (${\text{Cu}}_{I}^{•}$ or ${\text{Cu}}_{I}^{••}$) being a donor is schematically shown in Fig. 2A and results in an increase of the permittivity. This value, however, is not solely determined by the absolute concentration and charge of the particular species but also accounts for the chemical nature of the dopants and for the interactions with surrounding matrix and other dopants.

For example, replacing Al in SiO_2_:(Al,Cu)_1_ by same amount of Ga results in a lower permittivity of ε_rSiO2:(Ga,Cu)_ = 4.60 (compared to 6.81), because Ga forms a neutral defect complex. A direct correlation of the absorption band at 215-nm wavelength observed in Ga-doped SiO_2_ with increasing doping concentration suggests a defect complex with an associated negatively charged *E*′ center (*26*). Therefore, Ga only indirectly forms a different negatively charged defect center and thus creates different defects in SiO_2_ than aluminum with its AlO_4_^−^ configuration (*25*) as shown in Fig. 2B with its strongly bonded dipoles with Cu. Still, Ga has same oxidation state as Al and also leads to the same amount of negatively charged defect complexes, but with strongly differing dipolar interaction to dissolved Cu ions.

Dopants and matrix are also interacting with moisture. For example, whereas the slopes of permittivity versus *p*_H2O_ dependence for (Al,Cu)-doped samples are relatively steep (fig. S4), the slope for the (Ga,Cu)-doped sample is much flatter, indicating a weaker interaction of the matrix with moisture, due to chemical saturation effects induced by Ga doping. Therefore, by properly selecting the doping element, we can design a material, respectively a memristive cell, with stronger or weaker interactions/bonding between mobile and immobile defects (resulting in different transport properties, switching kinetics, and filament stability) and robustness against humidity and/or other external influences (*27*).

### Electrical double layer structure, capacitance, and electrochemical capacitance

The processes of mass (ions) and charge (ions/electrons) transfer during electrochemical reactions occur within the electrochemical double layer (EDL). The principle structure, thermodynamic, and kinetic conditions in the EDL significantly differ from these of the electrode and electrolyte. Within the double layer, strong gradients of both electrical and chemical potentials are persisting, whereas no such gradients exist within the metallic electrode and electrolyte phases for macroscopic samples. In nanoscale systems, double layers may overlap at conditions, fulfilled in most cases by memristive devices. Such an overlap results in a field-accelerated kinetics (redox reactions and ion migration) and can electrostatically hinder or enhance the redox reaction rate and distribution/movement of charged species, thus modifying the device kinetics and stability of ON, OFF, and intermediate states. Thus, thickness and structure of the EDL are of special importance for the switching kinetics, filament stability, and dynamics. Details on the principle structure and importance of the double layer for ReRAM systems are provided in Supplementary text S4.

**Fig. 3:**
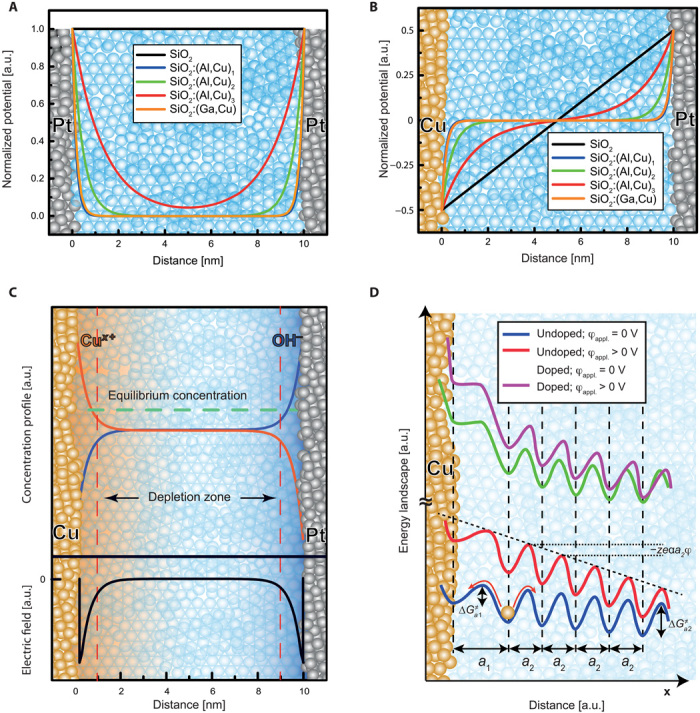
Potential and energy distribution in nanoelectrochemical memristors. (**A**) Symmetric cell with Pt electrodes. (**B**) Asymmetric cells with Cu and Pt as electrodes. Details on the calculation of the Debye length and the corresponding values are given in Supplementary text S3. (**C**) Charge separation and formation of ion-enriched and depleted zones in asymmetric memristive devices with two mobile-charge species and the corresponding electric field distribution [or SiO_2_:(Al,Cu)_2_]. In the particular case, the electric field drops only within the small zones of higher charge concentration. In case of pure material, the field will drop across the entire film thickness [see (B)]. (**D**) Schematic sketch of the energy landscape for pure and doped samples with and without applied external voltage. The difference between doped and pure SiO_2_ is presented by the change in the principle shape of the energy potential curve. Charge separation can additionally occur under applied external bias in cases that jump distances *a*1 and *a*2 and/or activation energies Δ*G*^≠^*_a_*_1_/Δ*G*^≠^*_a_*_2_ for redox reactions and transport, respectively, differ. a.u., arbitrary unit.

In Fig. 3, shown is the electric potential/field distribution for a symmetric Pt/SiO_2_/Pt and an asymmetric Cu/SiO_2_/Pt stack as a function of the EDL thickness in pure and doped SiO_2_. For the calculation of the space-charge layer thickness, two factors were accounted—the concentration of charges and the change of the dielectric constant caused by doping with foreign elements. For undoped SiO_2_, the Debye length is calculated λ_*D*,undoped_ = 69 μm, and the electric potential cannot relax over the oxide thickness (10 nm). The double layers overlap and the electric field drops linearly over the entire film thickness. For higher dopant/impurity concentrations, λ*_D_* becomes smaller and the potential/field distribution changes to lastly take its classical form.

The electrochemical gradients and potential distribution profiles in memristive cells are determined by intrinsic and extrinsic factors. Intrinsic factors are considered the number of charges and dielectric properties of the material. Extrinsic factors are considered a chemical asymmetry of the electrodes, externally applied pressure, or introduction of volatile impurities (e.g., moisture/protons). For example, Cu/SiO_2_/Pt devices have chemically asymmetric electrodes, resulting in an internal electromotive force Δφ_int_ (nanobattery effect) that may reach over 400 mV (*15*). This internal Δφ_int_ leads to a charge separation within the EDL and formation of charge (ion) clouds of opposite sign at electrode/electrolyte interfaces and the formation of a depleted intermediate region (more insulating) as shown in Fig. 3C. In case of a single mobile-type charge, one of the interfaces will be enriched and the other is depleted of charge. Similar considerations apply to valence change memories (fig. S6) or other nanoionic/nanoelectronic devices, sharing common electrochemical fundamentals (*21*).

The internal structure and ionic rearrangements make the cell similar to electrochemical capacitor, where the capacitance value depends on Δφ_int_, the dielectric constant of the electrolyte material, its thickness, and the initial concentration of mobile charges *N*_0_. Impurities/dopants contribute to changing the classical capacitance, whereas the mobile (volatile) impurities/dopants determine the electrochemical capacitance. To compensate the internal voltage, concentration *N* profiles of the mobile charges are induced, as shown in Fig. 3C, following the Poisson-Boltzmann exponential dependence (*28*)

$$N=N_{0}\mathrm{na}\text{exp}(\frac{\mathrm{ze}}{k_{B}T}\Delta \varphi_{\text{int}})$$ (1)

with *a* being the jump distance corresponding to the distance between two monolayer planes of the solid electrolyte, and *n* is the total number of these planes (*na* is giving the thickness of the oxide film *d*), *z* is the charge number, *e* is the electron charge, and *k*_B_ and *T* are the Boltzmann constant and temperature, respectively.

Thus, both volatile and nonvolatile impurities/dopants strongly influence and determine the structure of the space-charge layer and its capacitance, including the classical and electrochemical capacitances. These properties, in turn, are responsible for the switching performance and neuromorphic functions.

### EDL enhanced redox reactions and transport

These specifics of nanoscale cells have decisive influence on the switching kinetics. Depending on the concentration of impurities/dopants, three boundary cases can be distinguished. (i) Low concentration of charge carriers, where the EDL thickness is comparable to the solid electrolyte film. Depending on the sign of Δφ_int_, it will be either added (accelerating effect) or subtracted (suppressing effect) to/from applied bias during device operation, thus significantly changing the reaction rate and switching time. This leads to field-enhanced reaction kinetics (*29*, *30*) and transport (*31*)

$$i=\text{N ν ze exp}(-\frac{\Delta G_{a}^{\neq }}{k_{B}T})\text{exp}(-\frac{\text{α aze}}{k_{B}T}\frac{\Delta \varphi_{\text{a ppl}}}{d})$$ (2)

Combining Eqs. 1 and 2, we obtain for the reduction process that not only depends on the applied voltage Δφ_appl_ but also includes Δφ_int_

$$\begin{array}{c} i=\text{N ν ze exp}(-\frac{\Delta G_{a}^{\neq }}{k_{B}T})\text{exp}(-\frac{\alpha \text{aze}}{k_{B}T}\frac{\Delta \varphi_{\text{appl}}\pm \Delta \varphi_{\text{int}}}{d})\\ =i_{0}\text{exp}(-\frac{\alpha \text{aze}}{k_{B}T}\frac{\Delta \varphi_{\text{appl}}\pm \Delta \varphi_{\text{int}}}{d}) \end{array}$$ (3)

with *i* being the current density, and Δ*G*^≠^_*a*_ is the activation energy, α is the transfer coefficient, and *i*_0_ is the exchange current density.

Equation 3 can be formally applied to both charge-transfer and transport-limited kinetics. The difference will be only in the particular values for the activation energy $\Delta G_{a}^{\neq }$, the jump distance *a*, and the resulting charge-transfer coefficient α. These factors can be alone a reason for formation of regions with locally uncompensated charges (Supplementary text S5).

(ii) High concentration of impurities/doping. In this case, the EDL thickness is smaller compared to the thickness of the switching film (see Fig. 3, A and B for high concentrations), and in the general case, we do not expect field (EDL) enhancement. The classical Buttler-Volmer and diffusion equations apply for the reaction kinetics and charge. Field acceleration effect may occur only at much higher applied voltages, depending on the film thickness.

(iii) An intermediate case as exampled by SiO_2_: (Al,Cu)_3_ in Fig. 3 (A to C). This case is most complex. Depending on the presence and magnitude of Δφ_int_, charge separation will occur with formation of EDL structure similar to electrochemical capacitance (Fig. 3C). This will lead to formation of zones enriched depleted of mobile charge carriers, leading also to inhomogeneous EDL acceleration and kinetics.

### Effects of volatile and nonvolatile impurities/dopants on EDL structure

Volatile (mobile) dopants such as H^+^, Ag^+^, Cu^+^, O^2−^, and their compensating defects such as electrons, holes, oxygen vacancies, etc can be accumulated or depleted in the device by exchange with the environment, electrode reactions (for example, Cu dissolution or reduction, oxygen evolution/incorporation, etc), temperature exposure, and/or chemical interactions. In general, accumulation of volatile impurities/charges leads to a shift from the boundary case of low concentration (i) to the boundary case with high concentration (iii). This process is related to the elimination of the EDL acceleration effect (slower reaction rate and transport), short circuits and higher power consumption, and last, to device degradation.

Nonvolatile defects have the advantage that they do not accumulate or deplete. Adding immobile dopants to SiO_2_, i.e., Al or Ga, results in electrostatic interactions between the dopant(s) and mobile ions. Depending on the chemistry of the elements and the charge/size of the ions, the bond strength will be weaker or stronger. These bonds lead to a less pronounced charge separation to changes in the potential/field distribution and all kinetic parameters but especially, the activation energy for transport, increased by the energy for breaking the bond to the doping element (Fig. 3D). In general, nonvolatile dopants change the capacitance of the cell (in addition to the electrochemical capacitance) and lower the EDL acceleration and diffusion/migration rate but stabilize the filament.

Thus, on the basis of the analysis of the impurity/dopant-dependent EDL structure, one should expect faster kinetics for pure materials and slower kinetics for doped materials, where the dopants increase the activation energy for reaction and transport. During device operation, volatile dopants may accumulate (depletion is less probable), which will lead to degradation of the initial behavior.

### Impurity/dopant-controlled switching kinetics

**Fig. 4:**
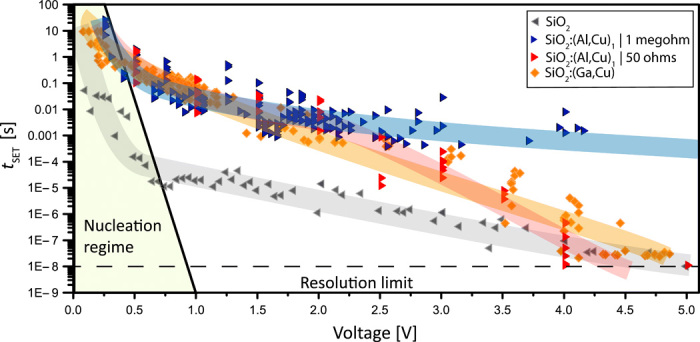
SET kinetics for SiO_2_-based devices. In the low-voltage regime, the SET kinetics are limited by the nucleation time, and the slope of the kinetics is steep. For higher voltages, the time-determining mechanism is strongly dependent on the doping and its influence on the switching properties. The undoped device (gray) show the typical trend for an ECM switching kinetic with a pronounced steep nucleation regime followed by a flatter slope representing a limitation by the ion movement and/or redox processes (*45*). For the highly doped SiO_2_:(Al,Cu)_1_, in devices with the highest permittivity, the kinetics are much slower and additionally limited by the RC time given by the high capacity and the shunt resistance in the setup. With a 1-megohm resistance, the switching speed is limited to times of around 1 ms at 5 V (blue); see also Supplementary text S6. When measuring with 50-ohm impedance, the kinetics are similar up to 2 V. Above 2 V, the kinetics with the low impedance are becoming faster than with high impedance. The SET kinetic shows a plateau at voltages lower than 2 V. We account this to the stronger interaction between the doped SiO_2_ (especially the AlO^4−^ clusters) and the Cu^2+^ ions (formation of defect associations; see also Fig. 2) involved in the switching.

The switching kinetics and the type of device functionality can be predetermined and/or adjusted by the type/chemistry and levels of doping. Undoped SiO_2_ shows the fastest SET times, being orders of magnitude lower compared to doped samples. In Fig. 4, pronounced nucleation regime and overlapping charge-transfer and diffusion-limited kinetics can be observed. By reducing the SiO_2_ film thickness to 3 nm, we achieved *t*_SET_ = 1.4 ns to be the fastest switching time for ECM devices reported up to now (fig. S8). The reason for this fast kinetics is the purity of the material. As predicted from Fig. 3, for pure SiO_2_, the space-charge layers overlap, and the kinetics is exponentially enhanced by the electric field within the entire film thickness. Because of the low *N*_0_, there is no strongly pronounced charge-separation effect, and the electric field is almost homogeneously distributed within the SiO_2_. No electrostatic interaction with doping elements is present. The external voltage is in this case superimposed to the in-built voltage, resulting in extremely fast switching time.

Highly doped samples show much slower kinetics. The nucleation-limited regime for SiO_2_:(Al,Cu)_1_ devices is subtle, followed by a plateau in the voltage range 1 to 2 V. We propose that this plateau is caused by the additional energy that is needed to break the bond between copper and AlO_4_^−^ clusters. Only after this activation energy is overcome that Cu ions can further respond to the voltage signal and move/switch faster. This effect is highlighting the importance of the interactions between incorporated dopants for the device kinetics.

The region above 2 V is characterized by a mixed charge transfer and diffusion limitation, logarithmically dependent on time. Last, for voltages above ~4 V, field effects are too strong and SET time for doped and undoped SiO_2_ becomes equal.

Samples with lower Al and Cu concentrations, i.e., SiO_2_:(Al,Cu)_2_ and SiO_2_:(Al,Cu)_3_, show SET kinetics transiting from low- to high-doping regime (fig. S9). Evidently, even small amounts of dopants and/or impurities have significant influence on the switching.

The results for SiO_2_:(Ga,Cu) are following the conclusions derived for the Al dopant, but because of weaker Ga-to-Cu electrostatic interactions, SiO_2_:(Ga,Cu) devices show lower permittivity compared to SiO_2_:(Al,Cu)_1_, leading to smaller capacities; but more importantly the copper ions can be easily separated from Ga ions, leading to a faster switching.

Volatile doping/impurity also strongly influences ReRAM kinetics. Incorporating moisture/protons in both ECM and VCM cells influences the switching, even if small amounts of the volatile dopant are present (*32*, *33*). As evident from the dielectric measurements, effects of volatile and nonvolatile dopants accumulate, and same influence was observed on the device performance. From a physicochemical point of view, there is no qualitative difference for the influence of the dopant incorporated within the electrolyte material, irrespective whether it is volatile or nonvolatile.

Thus, the concentration and chemistry of impurities/dopants are decisive for determining device behavior. From a fundamental perspective, they change the dielectric and ionic/electronic properties of the switching layer and determine the structure and thickness of the electrical double layer. These fundamental factors are practically essential and determine the device kinetics, filament growth, dissolution and stability, and related memory, selector, and neuromorphic functions.

### Impurity/dopant-controlled neuromorphic functionalities

Memristive devices are used to emulate neuromorphic functions, building artificial neural networks for “deep learning,” pattern recognition, signal processing, and brain-inspired spiking-based computation (*8*, *34*–*38*). Key feature is the ability to imitate synaptic plasticity, i.e., to learn/forget depending on the amplitude and frequency of input signal. In biological systems, this change (synaptic weight) relies on the modulation of cation (e.g., Ca^2+^, Na^+^, K^+^) conduction channels by electrical stimuli (*39*), being categorized in two time scales: STP and LTP (*40*). In STP, the change is volatile and the synaptic weight relaxes to its ground state. The LTP is nonvolatile and commonly represents the long-term memory function of a synapse. The transition between STP and LTP is fluent, depending on pulse amplitude, length of the pulses, length of pulse delays, and the history of synapse/device itself. A synapse/device should be able to be potentiated (SET to ON state) with positive pulses and depressed with negative pulses (RESET to OFF state). In the common praxis, the particular pulse train schema controls potentiation and depression.

Doping of the oxide is an alternative, parallel, and most efficient way to modulate the potentiation and depression processes and control the pulse/delay window by tuning permittivity and electrochemical capacitance.

**Fig. 5:**
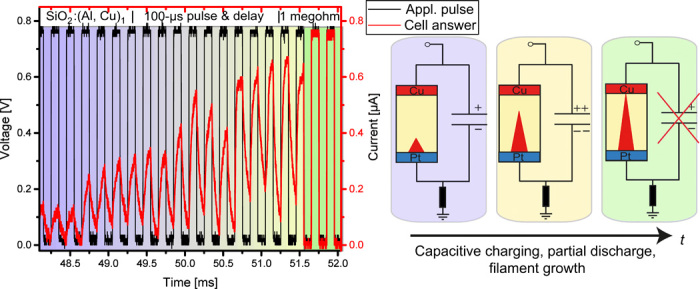
Potentiation of SiO_2_:(Al, Cu)_1_ memristive device. Red curve is the current response to the input voltage signal (black curve). As long as the switching filament has not short-circuited the device, the capacity is loaded and discharges in every pulse and following delay time (first region, marked in blue). In the second region (yellow), the filament continues to grow. As long as charge is stored in the capacity, a driving force for further filament growth is present. In the event of a fully formed filament (third region, marked in green), the capacity is shorted, and no further charging/discharging can be distinguished. The device’s capacity plays a significant role in the potentiation process for neuromorphic applications influencing STP component, which can not only learn and forget in short time ranges but also transition to long-term memory. The electrical circuit during potentiation is present at the right side in the figure.

In Fig. 5, a pulse train (0.8-V amplitude, 100-μs pulses, and 100-μs delay) and the corresponding response of SiO_2_:(Al,Cu)_1_ device are shown, divided into three regions. In the first region, the cell requires a certain number of potentiation pulse time to change its electrical conductivity. The incubation time of 48 ms is determined solely by charging and discharging the capacity, controlled by the type and amount of doping elements. Time and driving force are still not sufficient for Cu ions to migrate through the oxide film and form a filament.

In the second region, Cu-ion reduction starts at the Pt electrode, and the conductivity of the device consequently increases because of filament growth until short-circuit condition is reached. Here, because of oxidation, dissolution, and movement of Cu ions (volatile dopant), electrochemical capacitance is generated, and in addition, the classical capacitance is tuning the device properties. The final stage of the filament growth is evidenced in the *V* versus *t* dependence in Fig. 5 at 51.5 ms.

The dopant-controlled capacity plays an essential role for the learning mechanism. This capacity contains the contributions of both nonvolatile and volatile dopants, representing the classical and electrochemical capacitances. On one hand, the classical capacitance (nonvolatile impurities/dopants) slows down the kinetics of filament formation, but on the other hand, it is ensuring higher stability of the ON/intermediate states, allowing to play between these two parameters, which appears extremely important for neuromorphic applications. Moreover, during delay periods, the capacity (including electrochemical capacity, determined by the volatile dopants) acts as an additional voltage source and enhances the reduction rate of Cu^2+^ ions and can initiate formation and/or hinder the dissolution of a partially formed filament [nanobattery effect (*41*)].

**Fig. 6:**
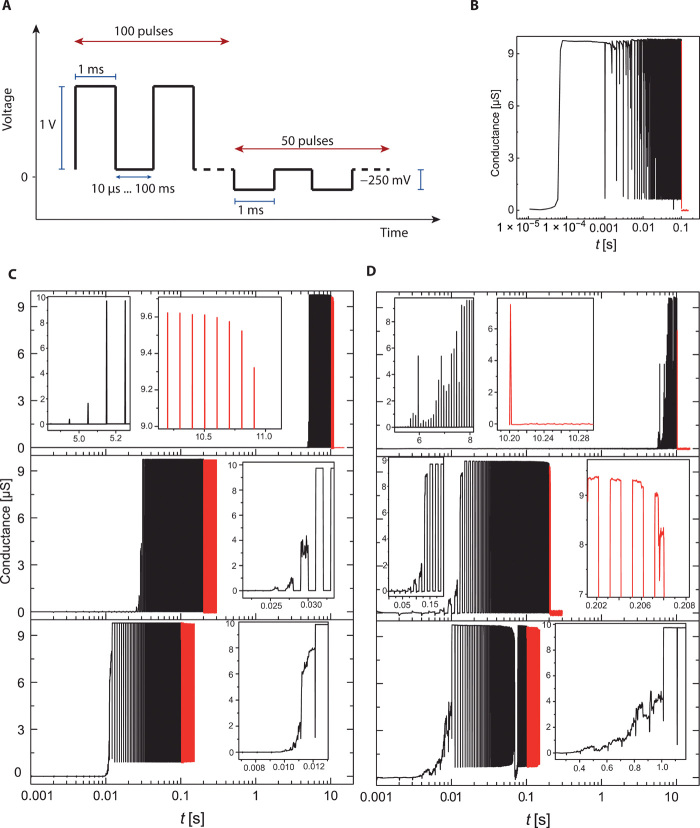
Potentiation and depression response of SiO_2_-based memristive devices. (**A**) The pulse scheme applied to the cells. (**B**) Undoped SiO_2_, (**C**) highly (Al,Cu)-doped SiO_2_, and (**D**) (Ga,Cu)-doped SiO_2_. The delay between the pulses was varied between 10 μs, 1 ms, and 100 ms (from bottom to top) to simulate different presynaptic firing rates. A resistor of 100 kilohms connected in series limits the current to protect the devices. Insets are given where applicable to show the potentiation (black) and depression (red) in more detail. In comparison, the undoped SiO_2_ shows no stable potentiation with the chosen series resistor. The depression is dependent on delay times during potentiation. Short delays form more stable filaments, whereas long delays form “softer” filaments, easier to depress. The potentiation and depression kinetics are mirroring the SET kinetics observed for same memory cells as shown in Fig. 4. The experiment demonstrates how device performance and functionalities can be adjusted to the materials’ design.

To demonstrate the influence of different nonvolatile dopants on potentiation and depression, we compared the responses of various devices applying pulse trains of 1-ms length with an amplitude of 1 V for the potentiation and −250 mV for the depression. The delay time was varied between 10 μs and 100 ms. Figure 6 shows the conductance of the undoped SiO_2_, the (Al,Cu)-doped, and the (Ga,Cu)-doped samples, respectively, where different delay times are sorted from 10 μs (lowest picture units) over 1 ms (mid) up to 100 ms. For undoped devices, no controlled potentiation is possible, as the device switches already during the first pulse. The formed filament is (depending on the selected current compliance/resistance) unstable and dissolves within a short time period. This fast filament formation can be used to shorten the required pulse length and repetitions or to lower the magnitude of the learning voltage to decrease the programming power. On the other hand, the fast dissolution (at higher series resistance) is not favoring LTP but makes these devices more suitable for fast-spiking neural networks or threshold applications, where the highly nonlinear nucleation regime is useful as filter for small pulse amplitudes. The internal driving force that enhances the fast self-driven RESET promotes this effect further.

Doped samples respond slower on potentiation (given by the larger capacitance and slower kinetics in low-voltage regime), providing more flexibility for control on neuromorphic applications. The switching kinetics for intermediate voltages (0.5 to 2.5 V) is almost identical, leading to a much higher immunity to small voltage differences in the input pulses. This also significantly improves the ability to control the potentiation (speed and STP/LTP transition) by the delay time over several orders of magnitude. In addition, the doping itself is enhancing the LTP function by stabilization of the metallic filament. The doping ensures initial concentration of Cu ions, lowering their gradient within the SiO_2_ matrix. As it can be seen in Fig. 6, the applied depression pulses of 10-μs delays are not sufficient to decrease the conductance for doped devices. At 1-ms delay time, depression is observed for SiO_2_:(Al,Cu)_1_ device, while SiO_2_:(Ga,Cu) device shows still constant conduction. For 100 ms, full depression is reached fast for SiO_2_:(Al,Cu)_1_, while the Ga-doped sample is showing only initial stages of depression. Thus, doping is a very powerful tool to tune the responses to voltage signals in neuromorphic type learning functionalities. This approach is in addition and independent to other learning approaches (e.g., by variation of the pulse duration, amplitude, and frequency) and can be used especially effective with variation of voltage amplitudes to find optimum LTP and STP behavior for the targeted applications.

## DISCUSSION

The presented results demonstrate that materials’ purity, the chemistry, and concentration of doping elements are essential factors in determining memristive functionalities. Using Cu/SiO_2_/Pt and Ta/Ta_2_O_5_/Pt as examples, we observed that impurities/dopants substantially change the device capacitance and switching/neuromorphic performance. By controlling the purity of the solid electrolyte and selecting the doping elements, one can adjust the permittivity, electrochemical properties at the interfaces and in the electrolyte, and related device characteristics. Dopants determine the structure, the overlapping of EDLs, and the extent of internal charge separation. They slow down the switching kinetics due to interactions with mobile ions but increases the stability of the ON and intermediate states. All these factors were identified as key elements for electric field–accelerated reaction kinetics and transport at the nanoscale.

Doping is also a powerful tool to tune the ability for short- and long-term potentiation by improving the retention of the conductive filament and the (electrochemical) capacity. Thus, we identify the nature of the relation between chemistry and concentration of impurities/dopants in the switching layer and device functionalities and offer powerful design directions to engineer memristive materials for the whole spectrum of memristive applications.

## MATERIALS AND METHODS

### Target preparation

The ultrahigh purity silica films were specially prepared for the project by Heraeus GmbH, using synthetic fused silica with highest purity and no residual OH^−^ content. For doped silica, we used powder with high–internal surface area that was impregnated with different metal-salt precursors of Cu, Al, and Ga (initial metal purity of at least 6N) of nominal doping concentrations in an aqueous solution. These slurries are subsequently dried to obtain doped powders using a rotary evaporator also made of fused silica to achieve maximum doping homogeneity with the lowest possible background impurity concentration. Doped powder samples are lastly melted in a high-purity vacuum oven process to obtain the doped glasses, which are finally cut and bonded into the final shape of the 2 inch sputtering targets. The obtained and tested targets with their corresponding dopant combinations are listed in table S1.

### Thin-film deposition and characterization

Radio frequency (RF) magnetron sputtering was used for thin-film deposition in an off-axis configuration with rotating substrate to obtain homogeneous layers. The process gas mixture (Ar/O_2_) and the substrate temperature were controlled to obtain dense SiO_2._ The optimum parameter set was found to be ≥150°C substrate temperature and 2-μbar process pressure with a gas flow of 9-sccm Ar and 1-sccm O_2_. The sputtering power was set to 150 W.

The microstructure of the films was controlled by scanning electron microscopy (fig. S1), and for the rate determination and density of the sputtered films, x-ray reflectometry was used (fig. S2). The component distribution was monitored by secondary ion mass spectrometry depth profiles (fig. S2).

### Device preparation and characterization

The tested samples were prepared in two different geometric configurations. For the capacitance measurements, 50 nm of the (doped) SiO_2_ were sputtered with optimum parameter sets on a Si/SiO_2_/Ti/Pt substrate as planar bottom electrode (30-nm Pt). A photolithography step followed by electron-beam evaporation of 30-nm Cu and DC sputtering of 30-nm Pt to prevent excessive oxidation and finalizing lift-off step were performed to structure top electrodes between 25 μm by 25 μm and 1 mm by 1 mm.

For the kinetics and potentiation/depression measurements, we used a microcrossbar configuration with 5 μm by 5 μm junction size on Si/SiO_2_/TiO_2_ substrates. Photolithography-assisted structured platinum electrodes were DC sputtered and subsequently covered with 10 nm of the to-be-tested SiO_2_/dopant compositions via RF sputtering. The bottom electrodes were kept partially open by another lithography step. Last, a last lithography-assisted e-beam evaporation of 30-nm Cu and subsequent DC-sputtered Pt top electrodes completed the sample preparation.

The permittivities ε*_r_* were determined from capacitance measurements with an HP 4284A LCR meter. The voltages were set to 0 V DC and 20 mV AC with a frequency of 100 kHz. The permittivities were calculated according to $C=\varepsilon_{0}\varepsilon_{r}\frac{A}{d}$, with the capacity *C*, the vacuum permittivity ε_0_, the electrode area *A*, and the thickness of the oxide *d.* The ε*_r_* values were determined in air [≈35% relative humidity (RH), vacuum (*P* < 1 × 10^−4^ mbar corresponding to 0% RH) and wet nitrogen (>90% RH)]. The concentration of OH^−^/H_2_O in the film was calculated by the empirical formula $\varepsilon_{s}=3.8073+2.72*10^{-22}\frac{{\text{cm}}^{3}}{\text{ion}}*N$, where *N* is the concentration of hydroxyl groups per cubic centimeter.

The switching kinetics was measured using a Wavetec 365 pulse generator and a Tectronix DPO 7254C Oscilloscope. The first channel of the oscilloscope was set to 50-ohm input impedance to record the pulse applied to the cell, whereas the second channel was set to either 50-ohm or 1-meghom input impedance as shunt resistor. The voltage drop over channel 2 corresponds to the current flow through the cell. The equivalent schema is provided in fig. S3. The potentiation measurements in Fig. 5 were performed with the same setup and 1-megohm shunt resistance to observe RC-time implications.

The potentiation and depression measurements in Fig. 6 were performed with a Keithley 4200 using two pulse measurement units (PMU, each 50-ohm internal resistance) and a 100-kilohm resistance as current compliance to protect the devices. The pulses were applied via PMU-1 to the top Cu electrode, while the second PMU recorded the current response.

### Calculation of the Debye length

The Debye lengths λ_D_ for the differently doped SiO_2_ films are calculated corresponding to

$$\lambda_{D}=\sqrt{\frac{\varepsilon_{0}\varepsilon_{r}k_{B}T}{\sum_{i=1}^{N}N_{A}e^{2}c_{i}z_{i}^{2}}}$$

with the vacuum permittivity ε_0_, the relative permittivity ε_r_, the temperature *T* = 293 and 15 K, the Avogadro constant N_A_, the elementary electric charge e, the ionic species concentrations *c_i_*, the corresponding charge number *z_i_*, and *N* being the number of ionic species. For the calculations, only the metallic ions are taken into account. The dielectric constant for each material has been taken from the dielectric measurements as shown in Fig. 1. The results are listed in table S2.

## Supplementary Material

aaz9079_SM.pdf

## SUPPLEMENTARY MATERIALS

Supplementary material for this article is available at http://advances.sciencemag.org/cgi/content/full/6/19/eaaz9079/DC1
