# Supplementary material for: Design of defect-chemical properties and device performance in memristive systems
Source: Sci Adv. 2020 May 8;6(19):eaaz9079. doi: 10.1126/sciadv.aaz9079 (PMC7272230; doi:10.1126/sciadv.aaz9079)
Supplement: aaz9079_SM.pdf [file aaz9079_SM.pdf]

Science Advances
AAAS

advances.sciencemag.org/cgi/content/full/6/19/eaaz9079/DC1

Supplementary Materials for

Design of defect-chemical properties and device performance in memristive systems

M. Lübben, F. Cüppers, J. Mohr, M. von Witzleben, U. Breuer, R. Waser, C. Neumann*, I. Valov*

*Corresponding author. Email: i.valov@fz-juelich.de (I.V.); christian.neumann@heraeus.com (C.N.)

Published 8 May 2020, *Sci. Adv.* **6**, eaaz9079 (2020)
DOI: 10.1126/sciadv.aaz9079

This PDF file includes:

Sections S1 to S8
Figs. S1 to S9
Tables S1 and S2
References

Supplementary Materials

S1. Experimental Methods

S1.1. Target synthesis and thin films

Table S.1. Sputter targets and its dopant concentrations.

Sputtertarget	Dopant concentration(s) [ppma]		remarks
SiO_2	-/-		99,9999995 % SiO_2
SiO_2:Cu	2080 Cu		
SiO_2:$(Al,Cu)_1$	14696 Al	652 Cu	
SiO_2:$(Al,Cu)_2$	1443 Al	45 Cu	
SiO_2:$(Al,Cu)_3$	142 Al	5 Cu	
SiO_2:(Ga,Cu)	8268 Ga	39 Cu	

Pure and doped silica sputtering targets prepared by Heraeus GmbH according to (29, 30).

S1.2. Thin Film Deposition and Control

In Figure S1 the principle influences of the sputter parameters (substrate temperature and gas composition) in microstructure can be seen. The sputtering power was set to 150 W. The substrate temperature and the processing gas composition have a huge influence on the microstructure of the sputtered films. While the SiO_2 films are relatively porous if prepared at low temperatures, high substrate temperature deposition leads to a densification and improved homogeneity. An even larger effect is observed after addition of 10 % oxygen to the processing gas as can be seen comparing Fig. S1 a) and d). The oxygen content leads not only to a tenfold reduction of the sputter rate (~15 nm/min down to 1,75 nm/min), but also for a significantly improved homogeneity and reduced roughness of the films. As a consequence we determined the optimum parameters for SiO_2 deposition to be at ≥150°C and a processing gas mixture of 90% Ar and 10 % oxygen.

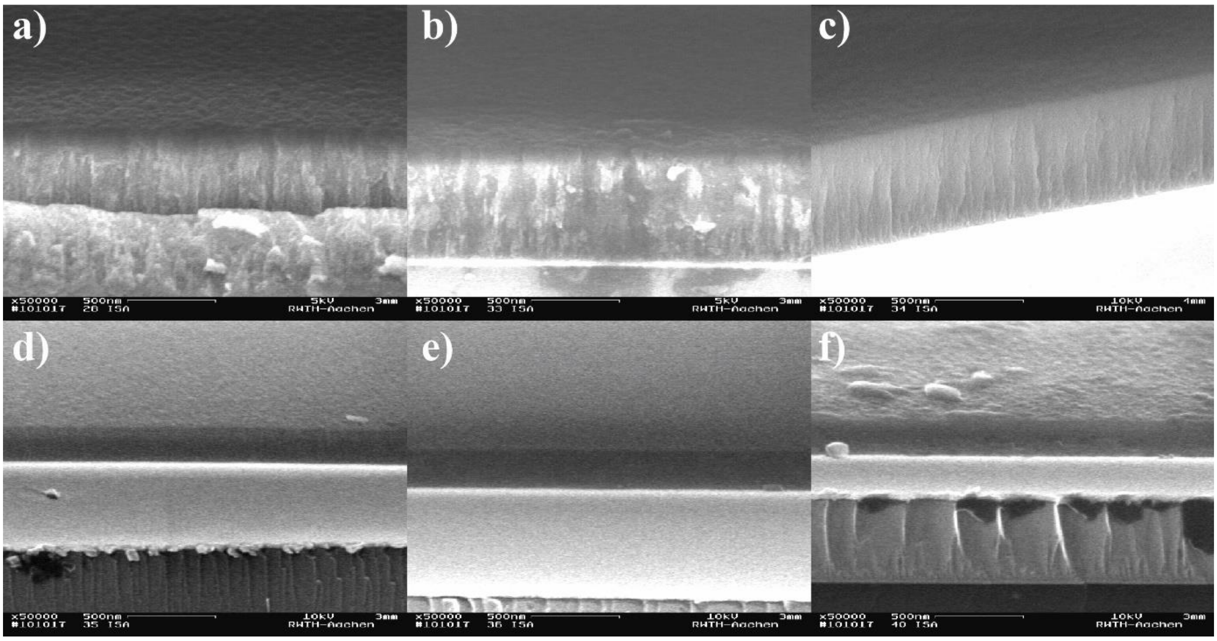

Figure S1: Scanning electron microscopy images of sputtered SiO_2 films on platinum and its broken edges at 50.000 magnification. Deposition at a) Oxygen-free conditions at room temperature. b) Oxygen free conditions at 100 °C substrate temperature. c) Oxygen free conditions at 200 °C. d) 10% oxygen gas mixture at room temperature e) 10% oxygen gas mixture at 100 °C f) 10% oxygen gas mixture and 200 °C.

The dense and pore-free deposition process of stoichiometric SiO_2 allows to avoid/suppress external influences, such as the uptake and release of oxygen and/or moisture. The increase of the film density was also verified by XRR measurements (Supplementary Figure S2A). For example, for an electron beam evaporated SiO_2 film of 30 nm thickness we extracted from the XRR a density of 1.95 g/cm³ -2.0 g/cm³ whereas for RF sputtered films used in this study we obtained densities of ~2.2 g/cm³ - 2.25 g/cm³ regardless of the amount of dopant in the target.

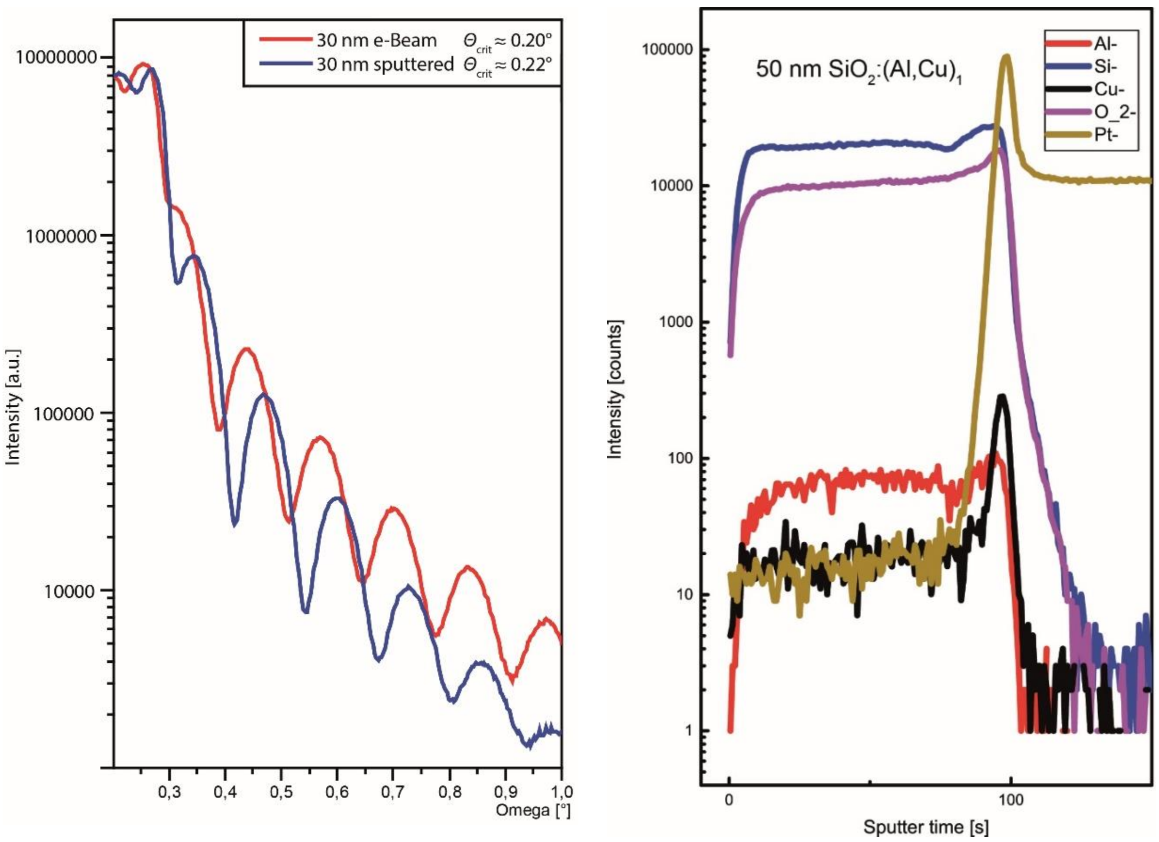

Figure S2: Thin film characteristaion. (A) Comparison of XRR measurements of electron beam evaporated (red) and sputtered SiO_2 films. The thicknesses of both films were determined to be ~30 nm, but have different critical angles Θ_{crit}, therefore having different densities. The densities are determined to be $\rho_{e\text{-}beam}$ = 1.95 g/cm³ -2.0 g/cm³ and $\rho_{sputtered}$ = 2.2 g/cm³ - 2.25 g/cm³, respectively. (B) SIMS depth profile of 50 nm sputtered SiO_2:(Al,Cu)$_1$ on platinized Si wafer substrate. The intensity of aluminum and copper mass signals is constant over the entire film thickness, verifying a homogeneous doping of the deposited film.

The homogeneity of the compositions of the sputtered films were verified using secondary ion mass spectrometry (SIMS) depth profiling. An exemplary SIMS measurement of a highly doped SiO_2:(Al,Cu)$_1$ film is shown in Supplementary Figure S2 B. The intensities of all elements are constant throughout the whole film thickness.

S1.3. Measurement Setup for Switching Kinetics.

The choice for the input impedances of 50 Ω and 1 MΩ was made because of the easy setup and low influence of parasitic capacities (short cables and pulling the shielding of the cables on same potential right after the device). To protect the cells from irreversible damages, 1 MΩ input impedance of the oscilloscope was used. However, the high input impedance can hinder the investigation of the underlying physical processes (high RC time), and therefore we performed in parallel measurements with 50 Ω shunt resistance. Using 50 Ω input impedance the setup allows for a small RC time, and the internal capacitance of the devices is quickly saturated (for example for $\varepsilon_r = 6$ the time is ~ 6.10^{-12} s) and the electrochemical processes and filament formation can further proceed. Whereas, when measuring with 1 MΩ input impedance, the RC time is drastically increased, the capacitance is loaded much slower (130 ns for same device), meaning that there will be a much longer time shift (voltage divider – equation S2) before switching processes can start.

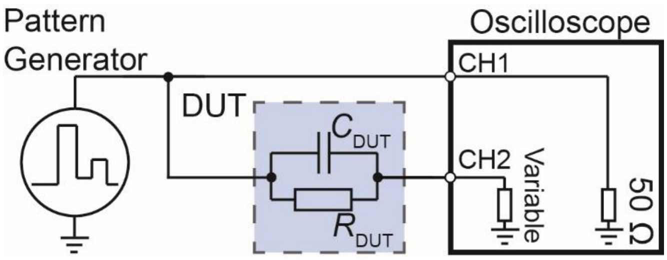

Figure S3: Measurement setup for the SET kinetics determination. The pulse generator sends a rectangular pulse to the device simultaneously measured at a 50 Ω terminated channel (CH1) of the oscilloscope. The device response is measured at an either 50 Ω or 1 MΩ terminated second channel as converted voltage drop over the shunt resistor corresponding to the current flowing through the device. The SET time was determined to be the time difference between the rising edges at 50 % height.

S2. Effects of moisture and materials' density.

The sensitivity of determination of dopant concentration by permittivity measurements is high. As it can be seen from the inset of Supplementary Figure S4 A even concentrations of 1 ppm to 20 ppm range can be effectively detected by this method.

In SiO_2 one can consider two different type of extrinsic defects – volatile and non-volatile. Protons and in general moisture as well other gases are considered volatile because in vacuum, in dry atmospheres and/or upon annealing these components leave the matrix. In contrary, Ga, Al and Cu remain in SiO_2. Of course, both type of defects can co-exists and each component present in the matrix will contribute to the change of the dielectric properties, irrespective whether these defects can potentially escape or not. Moreover, different dopants can also interact with each other.

Supplementary Figure S4 B shows the change in dielectric permittivity as a function of non-volatile dopant concertation at conditions where the uptake of moisture in SiO_2 is close to zero (e.g. in vacuum).

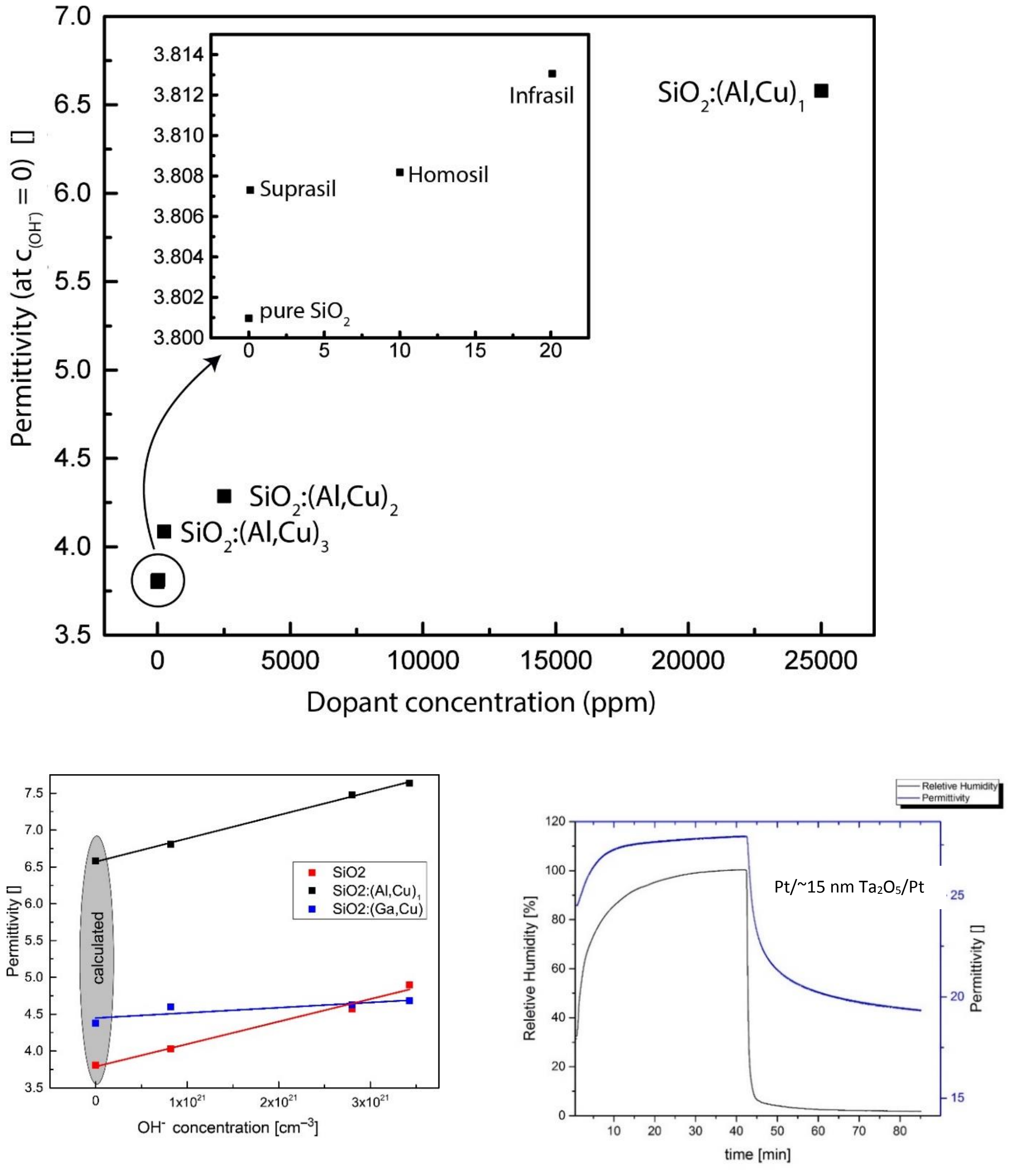

Figure S4. Permittivity as a function of dopant concentration for moisture free samples. (A) The inset is showing a zoomed region with the permittivity for industrial SiO_2 qualities Suprasil, Homosil and Infrasil with very low dopant concentrations, and ultra-pure SiO_2. (B) Comparison of the permittivity dependence of hydroxyl concentration for pure SiO_2, highly doped SiO_2:(Al,Cu)1 and doped SiO_2:(Ga,Cu). (C) Change of permittivity of Ta_2O_5 film in a symmetric Pt/Ta_2O_5/Pt device as a function of the relative humidity.

The permittivity increases linearly with increasing the non-volatile dopant concentration.

Incorporation of moisture and/or OH^--ions increases drastically the permittivity as water itself has a large ε_r value of 78-80 at room temperature (OH- and immobilized water can have a permittivity between 4 to 80, corresponding to the degree of immobility (46)), whereas defect free SiO_2 (e.g. Suprasil-W from Heraeus GmbH) has a ε_r value of 3.8073 at 300 K (26, 27). Because of the different electron affinities of the foreign dopants and/or defects, stronger or weaker (compared to silicon and oxygen) interatomic electrostatic interactions are induced. These interactions lead to formation of dipoles, influencing the permittivity and capacitance of the electrolyte film.

The ε-values were determined at 100 kHz in air (≈ 35 % relative humidity (RH)), vacuum (p < 1e-4 mbar corresponding to 0 % RH) and wet nitrogen (> 90 % RH). The concentration of OH^-/H_2O in the film was calculated by the empirical formula (42):

$$\varepsilon_s = 3.8073 + 2.72 * 10^{-22} \frac{cm^3}{ion} * N \qquad \text{(S1)}$$

where N is the concentration of hydroxyl groups per cm^3. Thus, we have measured for pure SiO_2 under vacuum conditions a permittivity of ε = 4.03, that is corresponding to 8.2×10^{20} cm^{-3} residual hydroxyl groups.

The moisture dependence of the film permittivity of undoped, (Al,Cu) doped and (Ga,Cu) doped samples is shown in Supplementary Figure S5. The undoped and (Al,Cu) doped films have a similar increase in permittivity with higher OH^- concentration levels, while the (Ga,Cu)-doped films have a flatter slope of the linear relation increase, indicating weaker electrostatic interactions between the incorporated water and the thin film.

Moisture is not only changing dielectric properties but is also an essential factor for the electrode processes, as it can undergo the required counter electrode reactions at the counter electrode interface. This process is essential for the oxidation of the active electrode material in ECM (13, 32) or the incorporation of oxygen in VCM (33) systems. The counter redox reaction can also influence the switching performance as rate-limiting step.

We identified nanoscale porosity as one of the crucial factors, influencing the uptake of moisture. For example, RF-sputtered samples of pure SiO_2 used in this study have permittivities ε_r ranging from 3.8 (dry - calculated) to 4.9 (wet) which corresponds to OH^- concentration of 3.4×10^{21} cm^{-3} in the film. However, electron-beam evaporated samples (higher porosity, indicated by lower density) show a tremendous dependence of the permittivity varying between $\varepsilon_r \approx 5$ in vacuum and $\varepsilon_r > 12$ in moisture-saturated atmosphere.

For other material systems e.g. Ta_2O_5 the effect of absorption of moisture is similar as in the case of silica (Figure S4 C).

Increasing the humidity levels results in the increase of the film permitivitty.

S3. Calculation of the Debye length.

The Debye length was calculated using the equation:

$$\lambda_D = \sqrt{\frac{\varepsilon_0 \varepsilon_r k_B T}{\sum_{i=1}^{N} N_A e^2 c_i z_i^2}} \quad \text{(S2)}$$

However, during uptake of moisture and/or doping the permittivity also changes. Expression that is more adequate is substituting equation S1 to obtain:

$$\lambda_D = \sqrt{\frac{\varepsilon_0 (3.807 + 2.72 \cdot 10^{-22} \cdot N) k_B T}{\sum_{i=1}^{N} N_A e^2 c_i z_i^2}} \quad \text{(S3)}$$

Unfortunately, it is not providing a general possibility to calculate the Debye length for the cases of external doping and influence of both moisture and external doping, because the parameters of the linear equation differ, depending on the chemistry of dopant, the bonds it forms with the matrix, and as well depends on the interaction between the dopants. Of this reason, we have re-calculated the Debye lengths and the corresponding profiles (Figure 3) using the experimentally measured values (from Figure 1) for each material.

Table S2. Calculated Debye lengths.

**Sputter target**	**Debye length [nm]**
SiO_2	69000
SiO_2:Cu	0.23
SiO_2:$(Al,Cu)_1$	0.17
SiO_2:$(Al,Cu)_2$	0.42
SiO_2:$(Al,Cu)_3$	1.34
SiO_2:(Ga,Cu)	0.21

S4. Space charge layer and potential/filed distribution in ReRAM cells

Electrochemical double layer (EDL) has crucial importance for the reaction kinetics in electrochemical systems. The metal/electrolyte contact and resulting changes at the interface (EDL) are shown in Figure S5.

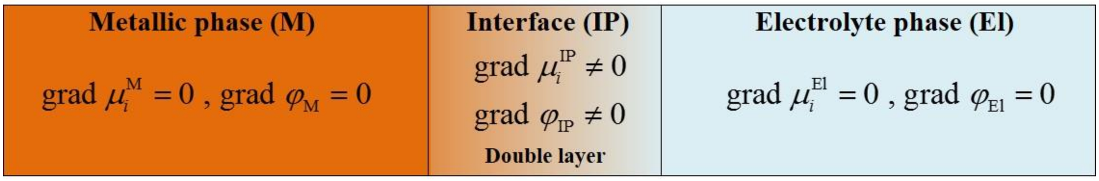

Figure S5. The structure of the Metal/Electrolyte interphase with formation of the electrochemical double layer (EDL).

For “classical” conditions there is no gradient of the chemical and electrical potentials neither within the metallic phase, nor within electrolyte phase. In contrast within the double layer strong gradients of both electrical and chemical potentials are created. Thus, the principle structure and conditions in the EDL significantly differ from electrode and electrolyte.

Important to note is that the processes of mass (ions) and charge (ions/electrons) transfer during electrochemical reactions occur exactly within the EDL (described in its classical form by the Butler-Volmer equation).

In the case of ReRAM cells we have often conditions where the double layers of the both electrodes overlap. In this case we have very strong influence of the local structure/charge distribution of the EDL on the reactions kinetics. This situation is depicted and discussed in Figure 3. In Figures 3A and 3B the black and red lines (pure and 200 ppm doped materials) we have overlapping of EDL. This leads to fast reactions and fast switching kinetics/time. Samples with higher impurity/dopant concentrations show properties closer to the "classical" systems.

Figure 3C shows the changes in the structure of the double layer (overlapping EDLs) in the case of chemically asymmetric electrodes. The internal electromotive force (nanobattery effect) creates a separation of the mobile charges and creation of a depletion zone. In case of immobile doping (e.g doping with Al and Ga or other dopants/impurities) the effect of charge separation will be less pronounced. Of this reason the applied external voltage accelerates the charges stronger in pure material (comparison blue/red line in Figure 3D) compared to materials with higher concertation of impurities/dopants. Additional information on the structure of the electrical double layer and its influence on the electrochemical kinetics can be found in the new ref. 31.

Figure 3D represents the case of high field reaction and transport where current depends exponentially on the electric field. Further details are provided in S6.

For valence change memory devices (VCM) the general picture is not changing compared to ECM cells. More specifically, in Supplementary Figure S6 is presented the situation for only one mobile ionic specie i.e. oxygen vacancies (in terms of Kroeger-Vink notation). In this case the vacancy accumulation will occur only at one of the electrodes (in this case Ta). The other

part of the oxide is depleted of oxygen vacancies. Of course, considering moisture or Ta^{5+} ions as additional ionic species, the profiles will take the form similar to that one shown in Figure 3.

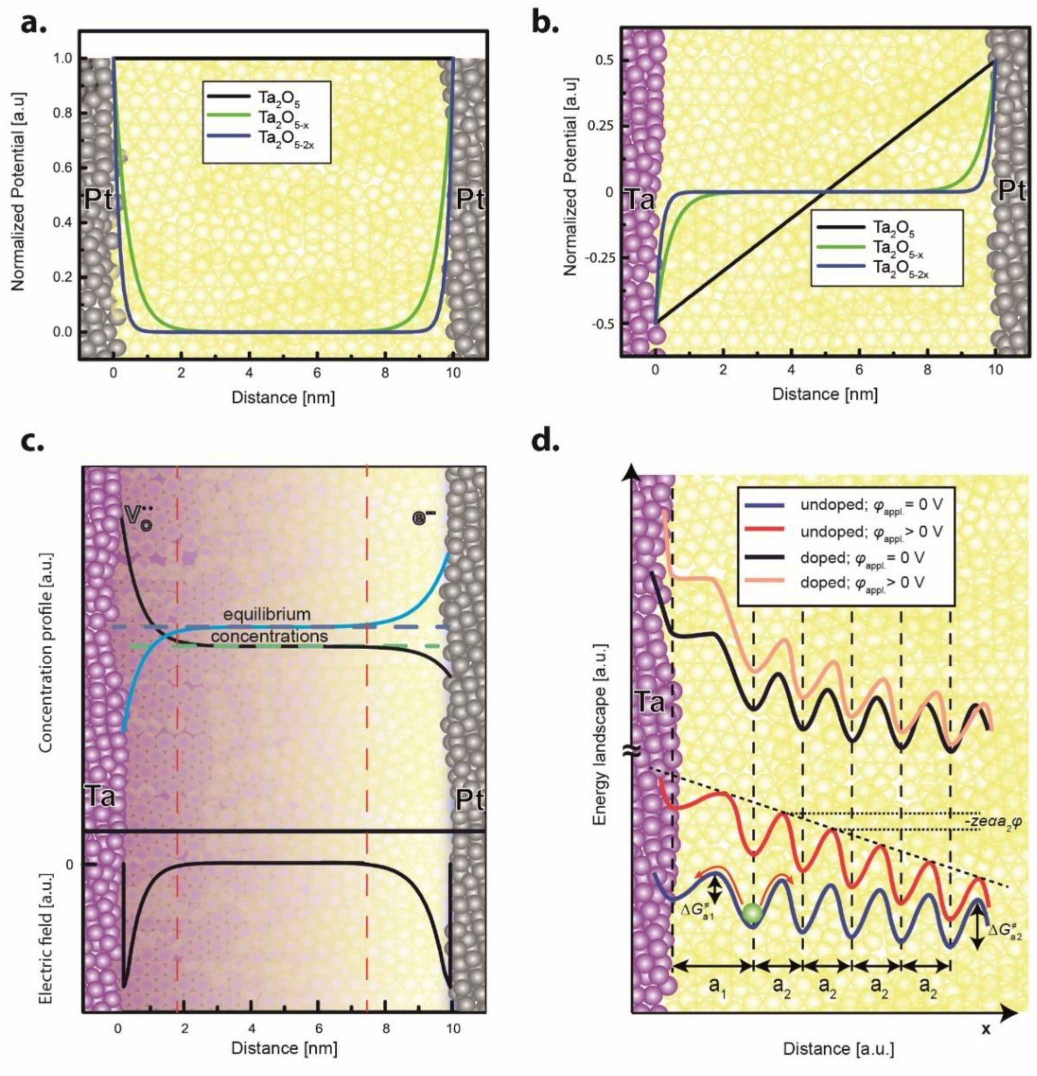

Figure S6. Potential and energy distribution in VCM cells. a) Symmetric cell with Pt electrodes b) Asymmetric cells with Ta and Pt as electrodes. The arbitrary values for the stoichiometry factor x are corresponding to 8N, 150 and 1500 ppm concentration of oxygen vacancies c) Charge separation and formation of vacancy enriched and depleted zones in asymmetric memristive devices with only one mobile ionic charge and the corresponding electric field distribution. In this case the electric field drops only within the small zone of higher charge concentration. In case of pure material the field will drop across the entire film thickness (see b)). d) Energy landscape for pure and substoichiometric samples with and without applied external voltage. Charge separation can additionally occur under applied external bias in cases that jump distances a_1 and a_2 and/or activation energies $\Delta G^{\neq}_{a1}/\Delta G^{\neq}_{a2}$ for redox reactions and transport respectively, differ.

As seen from the field/potential profiles the existence of enriched and depleted zones will have significant impact on the switching kinetics in terms of both thermodynamic and kinetics. In highly pure samples the ions experience the filed effect across the entire film thickness, whereas in samples with high concentration of charges the field drops only within the enriched part, leading to a slower kinetics.

S5. Effects of the parameters *a* and $\Delta G_{a}^{\neq}$ at applied external voltage.

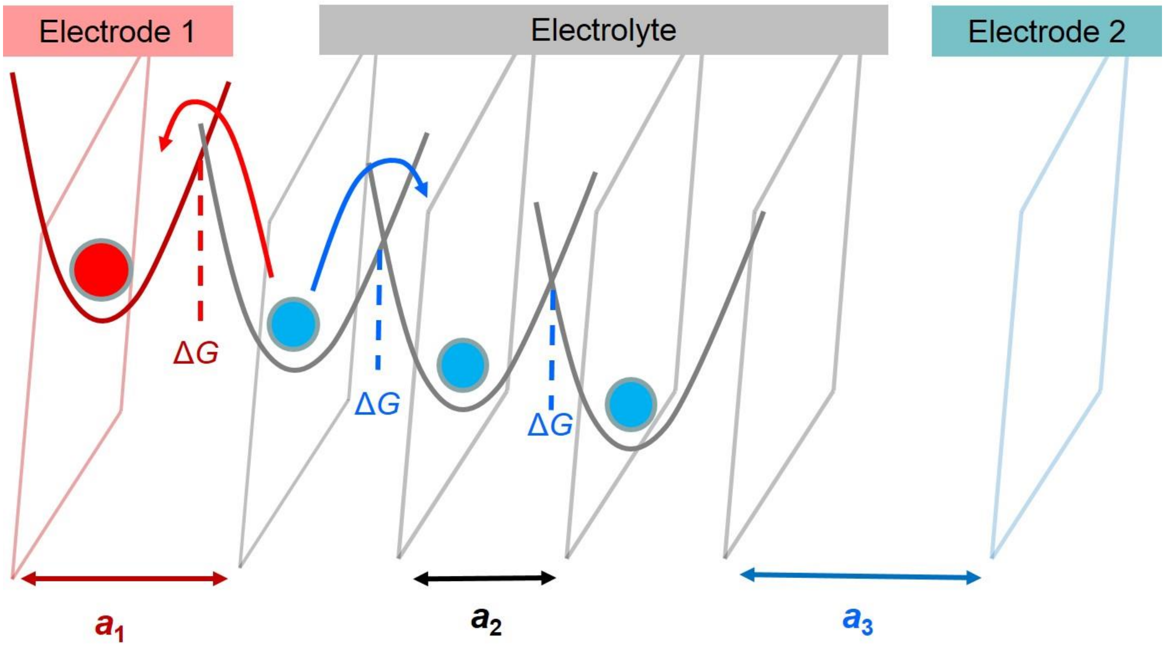

Figure S7. Energy barriers and distances during redox reaction (red) and ion transport (blue) in nanoscale systems subject to field acceleration. ΔG is the activation energy for the reaction (red) and transport (blue), and a is the distance between two positions (planes). For simplicity it is assumed that in the amorphous oxide there is only one (averaged) jumping distance for the transport.

Equation 3 (main manuscript) is formally representing field dependent redox reaction and transport, having own activation barrier and jump distance. The electrode redox reaction can be also regarded as a jump of an ion from the outset metal layer and first oxide layer (Figure S7). The jump distance and activation energy for redox reaction and transport in the general case is expected to deviate from each other, whereas these parameters should remain the same for the ion jumps related to transport within the film, for the case of pure material. Incorporation of

doping elements within the solid electrolyte can add a second jump distance/activation energy, particularly in the case that these are rate limiting.

In case of symmetric cells e.g. Pt/Cu:SiO_2/Pt $\Delta\varphi_{\mathrm{int}}$ is expected to be 0 and therefore at open circuit conditions less complex charge separation is expected. However, after external voltage $\Delta\varphi_{\mathrm{appl}}$ is applied, mobile charge carriers of opposite sign (and different mobility) will be attracted to the oppositely biased electrodes and charge separation will be induced. Considering the whole cell, the electroneutrality will be kept, but locally there will be regions enriched or depleted, respectively of mobile charges, resulting in different local electric field distribution.

In addition, variations in *a* and $\Delta G_{\mathrm{a}}^{\neq}$ can also lead to inhomogeneity in the electric field distribution. The jump distance *a* will or can be different within the same systems for the following cases: i) *a (redox)* and *a (transport). a (redox)* is the jump distance for the redox reaction i.e. this will be the distance from the metal electrode to the first layer of the oxide. This length is usually expected to differ from the jump distance for the ion during the transport in the oxide matrix *a (transport).* ii) In case of chemical or structural inhomogeneity of the oxide matrix there may be more than one jumping distance. For example, if we incorporate Al-ions that attract stronger the Cu-ions (compared to intrinsic defects). In such a case Cu slower transport/jump and longer jump distance *a (transport)* can be defined as from Al-to-Al ion. Structural defects such as dislocations, voids etc as well as nanocrystalline nuclei within amorphous matrix and/or agglomerations of different stoichiometry and/or composition will lead to same effect. In all these cases the activation energy is also expected not to be equal. Thus, upon application of external voltage charges can pile up at the defect locations and locally change the electric filed distribution.

S6. Influence of the protective resistance on the recorded SET time.

Analyzing and discussing the SET kinetics we have to consider one important external factor, i.e. the input resistance used to protect the devices from overshooting and damages. Using higher resistances (more reliable in protecting the device) on samples with high permittivity may result in SET time determined entirely from the time of charging the device capacitance. For example, measuring with 1 MΩ input impedance devices of 5 µm x 5 µm with a film permittivity of $\varepsilon_r = 6$, will result in a RC time of 130 ns. The voltage $\varphi_{internal}$ effectively driving the redox reactions and the ion drift is given by eq. S3.

$$\varphi_{int} = \varphi_{cap} = \varphi_{appl}(1 - e^{-t/\tau}) \qquad \text{(S3)}$$

with φ_{cap} being the voltage over the capacity, φ_{appl} the externally applied voltage, the time t and the RC time τ.

This effect was observed for highly doped SiO_2:(Al,Cu)$_1$ devices. As it can be seen in Figure 4 the SET time after exceeding voltage pulses of 2 V remained constant (blue region), limited be the RC time. The higher doping concentration and induced charge separation is leading to a formation of electrochemical capacitor (see Figure 3C). This additional capacitance (~ 15 µF/cm^2) increases the switching time in the saturation region to about 1 ms. To avoid this effect we used for these samples 50 Ω input impedance, lowering the estimated RC time to ~6.10^{-12} s, allowing to probe the actual SET kinetics of the devices.

S7. Approaching ultra-fast switching

To investigate the switching speed of the devices further below 10 ns timescale we also applied 10 ns pulses with a Picosecond PSPL2600C pulse generator and measured the current response with a Tektronix DPO73304D real time oscilloscope. The setup is similar to the one used in (47). To provide prober impedance matching at the contact pads the device was integrated into a coplanar waveguide structure. The applied voltage was set to 2 V. As the device has a high initial resistance the voltage of the applied pulse doubles over the devices stack resulting in 4V

at the device. In Supplementary Figure S8 the voltage at the device and the current response are shown. At the beginning of the pulse a small capacitive current can be observed which corresponds to the charging of the device. The SET event occurs approx. 1.4 ns after the maximum of the capacitive current.

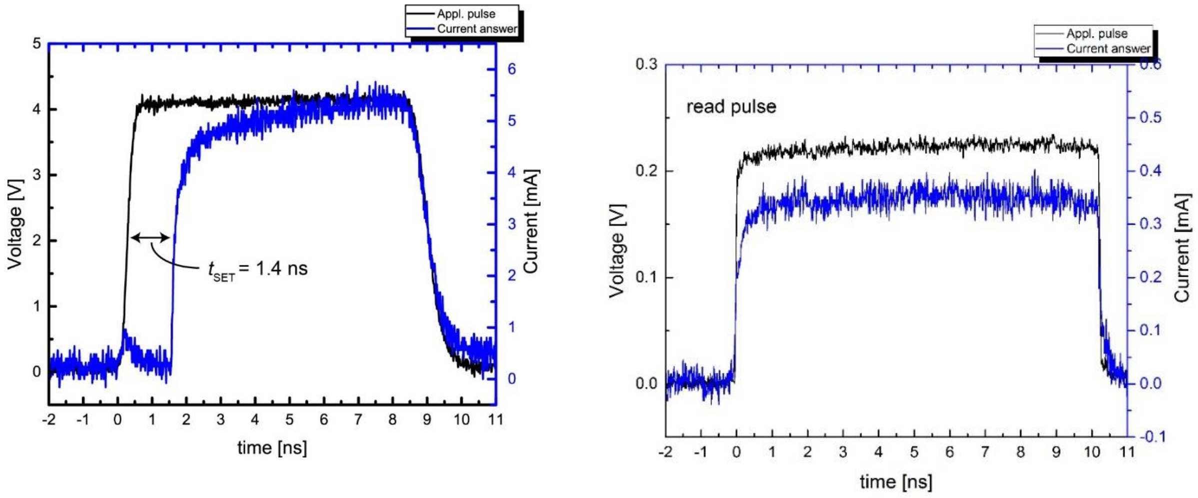

Figure S8: Demonstration of ultra-fast switching of an undoped device with 3 nm thin SiO2. (A) To investigate switching faster than 10 ns, this device was embedded in a coplanar waveguide structure and tested with a more sophisticated measurement apparatus including higher resolution and 50 Ω impedance matching. (B) Read pulse of the cell switched for 1.4 ns.

The following read pulse have confirmed the stability (non-volatility) of the ON state. It is also evidenced in Figure S8 B from the high ON state current (low resistance).

S8. Dopant concentration dependent kinetics.

The kinetics for all (Al,Cu) dopant concentrations (covering three orders of magnitude) are depicted in Supplementary Figure S9 A. In the low voltage regime (V < 1 V), the kinetics are almost congruent. For larger voltages the kinetics start to show different trends. The samples with the smallest dopant concentrations (SiO_2:$(Al,Cu)_3$) have a single slope up to the tested 5 V, independent on the input impedance. This indicates a weaker influence by the electrochemical capacitance and lower energy required to break the Al-Cu bonds. The medium doped SiO_2:$(Al,Cu)_2$ kinetics are also independent of the input impedance and the kinetics are

similar to the highly doped SiO_2:(Al,Cu)$_1$ (with 1 MΩ impedance) up to 3 V. However, for higher SET voltages, the kinetics of SiO_2:(Al,Cu)$_2$ are becoming steeper for voltages > 3 V.

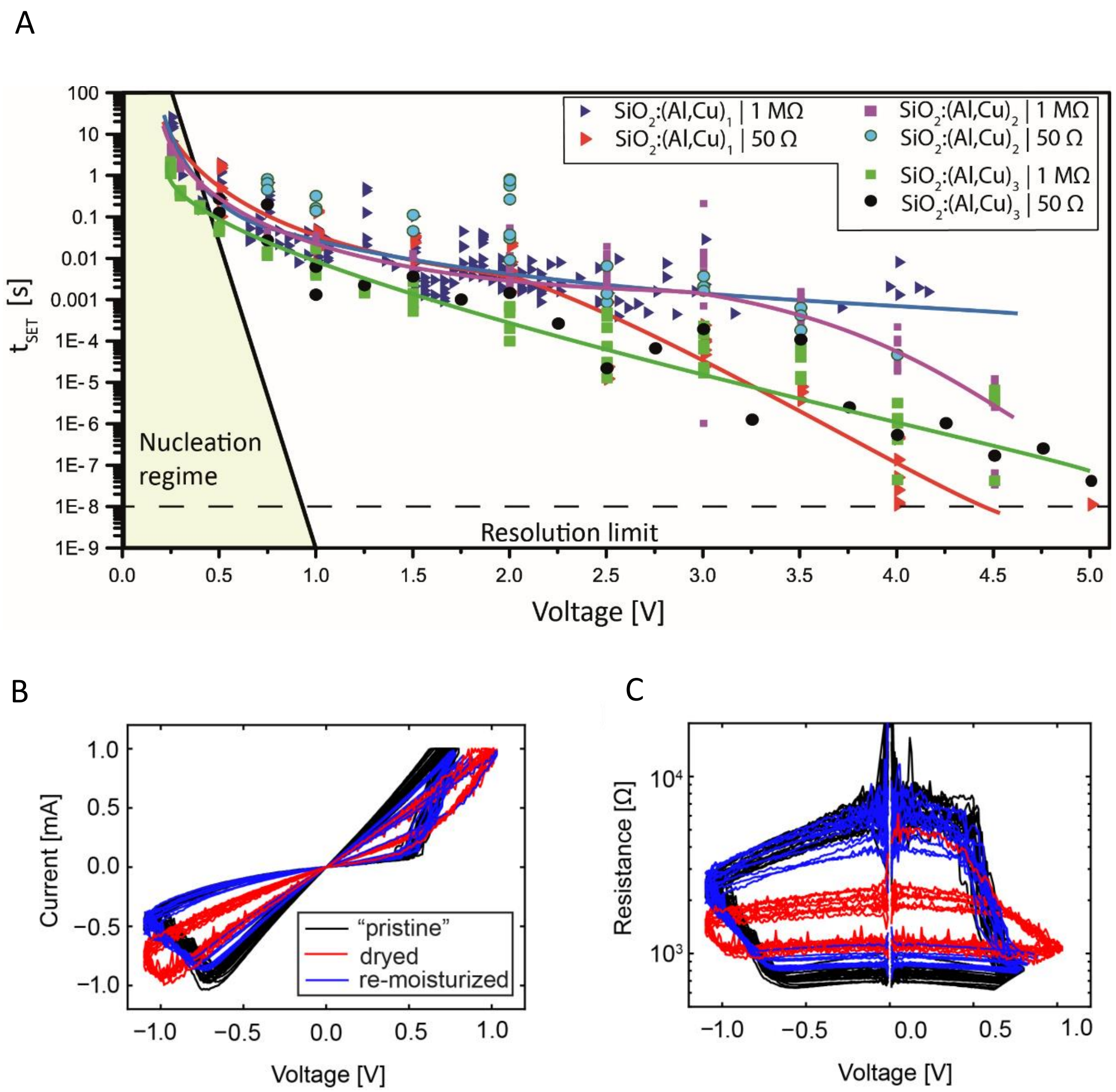

Figure S9. Kinetics of ECM and VCM devices as a function of non-volatile and volatile doping. (A) Kinetics measurements with (Al,Cu)-doped SiO_2 samples and different concentration levels over 3 orders of magnitude. (B) *I-V* sweeps for Ta/Ta_2O_5/Pt VCM device. The black curves correspond to initial conditions at 35% relative humidity (conditions in the lab). The red sweeps are performed after removing moisture from the film in vacuum heating at 200 °C for 1 h. The blue curves are the showing the sweeps after introducing ambient air in the chamber. All experiments were performed on the same device within the same setup, without breaking the experiment. The complete removal of moisture has been confirmed by SIMS measurements using D_2O saturated atmosphere to introduce Deuterium within Ta_2O_5 as a marker.

As it can be seen from Figure S9 B the switching performance of Ta/Ta_2O_5/Pt VCM device changes significantly when moisture is removed from the sample. Both ON and OFF resistances

change (OFF resistance more pronounced) and also the SET voltage is significantly increased. After introducing again air (Rh = 35%) in the chamber the *I-V* sweeps returned to their previous (initial) shape. This experiment clearly verifies that impurities/dopants have same influence on the switching kinetics in VCM as in ECM systems. (The influence of volatile dopants on the forming process and electrochemical behavior has been already demonstrated by us in series of papers for both ECM and VCM systems).

The effects of different impurities presented in the matrix are superimposed.

It is important to distinguish the experiment is this manuscript compared to those in Ag/SiO2 system reported in ref. 41. In the Nanotechnology paper the different concentrations that appear at the interfaces are solely result from different catalytic activity of the counter electrodes towards the counter electrode reaction. Therefore, higher catalytic activity results in higher reaction rate and lower switching time. The concentration in the SiO2 is constant (initially zero). The formation of increased Ag-concentration at the Ag/SiO2 interface begins after the voltage is applied and depends on the catalytic activity of the counter electrode. The thickness of the oxide film plays no direct role in this process. The proposed model cannot distinguish between surface and “bulk” concentration. Butler-Volmer equation is formulated for macroscopic systems where the concertation of ionic species in the bulk and at the surface is the same (charge transfer limited process). The level of development of the model is not allowing to incorporate the counter electrode reaction (coupled to the active electrode reaction) and making the distinction.

In the present manuscript the situation is principally different. The concentration of Cu in SiO_2 is different (systematically varied). An increased concentration of Cu ions at the interface as in Fig. 3C (and related charge separation) occurs only for the case of overlapping EDLs (low initial concentration of Cu in SiO_2 and/or lower thicknesses of SiO_2 film). This charge separation occurs before any voltage is applied. We do not change the counter electrode and

therefore no difference in the catalytic activity of the electrode is present. Here, (in contrast to Nanotechnology paper) the determining factor is the initial concentration of Cu in SiO_2 (we kept the thickness of SiO_2 constant).

REFERENCES AND NOTES

1. D. Ielm ini, H.-S. Philip Wong, In-memory computing with resistive switching devices. *Nat. Electro.* **1**, 333–343 (2018).

2. Y. Gonzalez-Velo, H. J. Barnaby, M. N. Kozicki, Review of radiation effects on ReRAM devices and technology. *Semicond. Sci. Tech.* **32**, 083002 (2017).

3. W. Chen, N. Chamele, Y. Gonzalez-Velo, H. J. Barnaby, M. N. Kozicki, Low-temperature characterization of Cu-Cu:Silica-based programmable metallization cell. *IEEE Electron Device Lett.* **38**, 1244–1247 (2017).

4. L. Nagarajan, R. A. De Souza, D. Samuelis, I. Valov, A. Börger, J. Janek, K. D. Becker, P. C. Schmidt, M. Martin, A chemically driven insulator-metal transition in non-stoichiometric and amorphous gallium oxide. *Nat. Mater.* **7**, 391–398 (2008).

5. S. Choi, S. H. Tan, Z. Li, Y. Kim, C. Choi, P. Y. Chen, H. Yeon, S. Yu, J. Kim, SiGe epitaxial memory for neuromorphic computing with reproducible high performance based on engineered dislocations. *Nat. Mater.* **17**, 335–340 (2018).

6. M. A. Zidan, J. P. Strachan, W. D. Lu, The future of electronics based on memristive systems. *Nat. Electron.* **1**, 22–29 (2018).

7. A. Serb, A. Khiat, T. Prodromakis, Seamlessly fused digital-analogue reconfigurable computing using memristors. *Nat. Commun.* **9**, 2170 (2018).

8. Z. Wang, S. Joshi, S. Savel’ev, W. Song, R. Midya, Y. Li, M. Rao, P. Yan, S. Asapu, Y. Zhuo, H. Jiang, P. Lin, C. Li, J. H. Yoon, N. K. Upadhyay, J. Zhang, M. Hu, J. P. Strachan, M. Barnell, Q. Wu, H. Wu, R. S. Williams, Q. Xia, J. J. Yang, Fully memristive neural networks for pattern classification with unsupervised learning. *Nat. Electron.* **1**, 137–145 (2018).

9. Z. Wang, S. Joshi, S. E. Savel'ev, H. Jiang, R. Midya, P. Lin, M. Hu, N. Ge, J. P. Strachan, Z. Li, Q. Wu, M. Barnell, G. L. Li, H. L. Xin, R. S. Williams, Q. Xia, J. J. Yang, Memristors

with diffusive dynamics as synaptic emulators for neuromorphic computing. *Nat. Mater.* **16**, 101–108 (2017).

10. Hans Schweickert Vorrichtung zur Gewinnung reinsten Halbleitermaterials fuer elektrotechnische Zwecke, *Deutsches Patent* DE1061593 B (1956).

11. A. J. Tan, M. Huang, C. O. Avci, F. Büttner, M. Mann, W. Hu, C. Mazzoli, S. Wilkins, H. L. Tuller, G. S. D. Beach, Magneto-ionic control of magnetism using a solid-state proton pump. *Nat. Mater.* **18**, 35–41 (2019).

12. T. Tsuchiya, K. Terabe, M. Ochi, T. Higuchi, M. Osada, Y. Yamashita, S. Ueda, M. Aono, In situ tuning of magnetization and magnetoresistance in Fe_3O_4 thin film achieved with all-solid-state redox device. *ACS Nano* **10**, 1655–1661 (2016).

13. D. Mantegazza, D. Ielmini, A. Pirovano, B. Gleixner, A. L. Lacaita, E. Varesi, F. Pellizzer, R. Bez, Electrical characterization of anomalous cells in phase change memory arrays. *2006 Int. El. Devices Meet.*, 1–4 (2006).

14. Z. Li, J. Lee, J. P. Reifenberg, M. Asheghi, R. G. D. Jeyasingh, H.-S. Philip Wong, K. E. Goodson, Grain boundaries, phase impurities, and anisotropic thermal conduction in phase-change memory. *IEEE Electron Device Lett.* **32**, 961–963 (2011).

15. S. Tappertzhofen, I. Valov, T. Tsuruoka, T. Hasegawa, R. Waser, M. Aono, Generic relevance of counter charges for cation-based nanoscale resistive switching memories. *ACS Nano* **7**, 6396–6402 (2013).

16. S. Tappertzhofen, R. Waser, I. Valov, Impact of the counter-electrode material on redox processes in resistive switching memories. *ChemElectroChem* **1**, 1287–1292 (2014).

17. D. Y. Cho, S. Tappertzhofen, R. Waser, I. Valov, Bond nature of active metal ions in SiO_2-based electrochemical metallization memory cells. *Nanoscale* **5**, 1781–1784 (2013).

18. A. Mehonic, M. Buckwell, L. Montesi, M. S. Munde, D. Gao, S. Hudziak, R. J. Chater, S. Fearn, D. McPhail, M. Bosman, A. L. Shluger, A. J. Kenyon, Nanoscale transformations in metastable, amorphous, silicon-rich silica. *Adv. Mater.* **28**, 7486–7493 (2016).

19. M. S. Munde, A. Mehonic, W. H. Ng, M. Buckwell, L. Montesi, M. Bosman, A. L. Shluger, A. J. Kenyon, Intrinsic resistance switching in amorphous silicon suboxides: The role of columnar microstructure. *Sci. Rep.* **7**, 9274 (2017).

20. A. Mehonic, A. L. Shluger, D. Gao, I. Valov, E. Miranda, D. Ielmini, A. Bricalli, E. Ambrosi, C. Li, J. J. Yang, Q. Xia, A. J. Kenyon, Silicon oxide (SiO_x): A promising material for resistance switching? *Adv. Mater.* **30**, e1801187 (2018).

21. I. Valov, W. D. Lu, Nanoscale electrochemistry using dielectric thin films as solid electrolytes. *Nanoscale* **8**, 13828–13837 (2016).

22. S. P. Thermadam, S. K. Bhagat, T. L. Alford, Y. Sakaguchi, M. N. Kozicki, M. Mitkova, Influence of Cu diffusion conditions on the switching of Cu-SiO_2-based resistive memory devices. *Thin Solid Films* **518**, 3293–3298 (2010).

23. C. Gopalan, M. N. Kozicki, S. Bhagat, S. C. P. Thermadam, T. L. Alford, M. Mitkova, Structure of copper-doped tungsten oxide films for solid-state memory. *J. Non Cryst. Solids* **353**, 1844–1848 (2007).

24. X. Guo, J. E. Jakes, M. T. Nichols, S. Banna, Y. Nishi, J. L. Shohet, The effect of water uptake on the mechanical properties of low-k organosilicate glass. *J. Appl. Phys.* **114**, 084103 (2013).

25. L. Skuja, M. Hirano, H. Hosono, K. Kajihara, Defects in oxide glasses. *Phys. Status Solidi C* **2**, 15–24 (2005).

26. C. Neumann, Optisches filtermaterial aus gallium-dotierten quarzglas, filterbauteil sowie verfahren zur bestrahlung mittels einer uv-strahlenquelle. European patent EP2414872B1 (2016).

27. C. Neumann, Resistive switching memory cell. European patent EP3208855A1 (2017).

28. P. Delahay, *Double Layer and Electrode Kinetics* (John Wiley & Sons Inc., 1965).

29. L. Young, *Anodic Oxide Films* (Academic Press, 1961).

30. A. Zaffora, F. Di Quarto, H. Habazaki, I. Valov, M. Santamaria, Electrochemically prepared oxides for resistive switching memories. *Faraday Discuss.* **213**, 165–181 (2019).

31. N. F. Mott, R. W. Gurney, *Electronic Processes in Ionic Crystals* (Oxford at the Clarendon Press, 1950).

32. M. Lüebben, S. Menzel, S. G. Park, M. Yang, R.Waser, I. Valov, SET kinetics of electrochemical metallization cells: Influence of counter-electrodes in SiO_2/Ag based systems. *Nanotechnology* **28**, 135205 (2017).

33. M. Lübben, S. Wiefels, R. Waser, I. Valov, Processes and effects of oxygen and moisture in resistively switching TaO_x and HfO_x. *Adv. Electron. Mater.* **4**, 1700458 (2018).

34. T. Ohno, T. Hasegawa, T. Tsuruoka, K. Terabe, J. K. Gimzewski, M. Aono, Short-term plasticity and long-term potentiation mimicked in single inorganic synapses. *Nat. Mater.* **10**, 591–595 (2011).

35. S. H. Jo, T. Chang, I. Ebong, B. B. Bhadviya, P. Mazumder, W. Lu, Nanoscale memristor device as synapse in neuromorphic systems. *Nano Lett.* **10**, 1297–1301 (2010).

36. P. M. Sheridan, F. Cai, C. Du, W. Ma, Z. Zhang, W. D. Lu, Sparse coding with memristor networks. *Nat. Nanotechnol.* **12**, 784–789 (2017).

37. C. Li, D. Belkin, Y. Li, P. Yan, M. Hu, N. Ge, H. Jiang, E. Montgomery, P. Lin, Z. Wang, W. Song, J. P. Strachan, M. Barnell, Q. Wu, R. S. Williams, J. J. Yang, Q. Xia, Efficient and self-adaptive in-situ learning in multilayer memristor neural networks. *Nat. Commun.* **9**, 2385 (2018).

38. Z. Wang, M. Rao, J.-W. Han, J. Zhang, P. Lin, Y. Li, C. Li, W. Song, S. Asapu, R. Midya, Y. Zhuo, H. Jiang, J. H. Yoon, N. K. Upadhyay, S. Joshi, M. Hu, J. P. Strachan, M. Barnell, Q. Wu, H. Wu, Q. Qiu, R. S. Williams, Q. Xia, J. J. Yang, Capacitive neural network with neuro-transistors. *Nat. Commun.* **9**, 3208 (2018).

39. B. A. Simms, G. W. Zamponi, Neuronal voltage-gated calcium channels: Structure, function, and dysfunction. *Neuron* **82**, 24–45 (2014).

40. A. Citri, R. C. Malenka, Synaptic plasticity: Multiple forms, functions, and mechanisms. *Neuropsychopharmacology* **33**, 18–41 (2008).

41. I. Valov, E. Linn, S. Tappertzhofen, S. Schmelzer, J. van den Hurk, F. Lentz, R. Waser, Nanobatteries in redox-based resistive switches require extension of memristor theory. *Nat. Commun.* **4**, 1771 (2013).

42. C. Andeen, D. Schuele, J. Fontanel, Effect of Oh^{-} on the low-frequency dielectric-constant of vitreous silica. *J. Appl. Phys.* **45**, 1071–1074 (1974).

43. Heraeus Data Sheet Quartz Glass for Optics-Data and Properties (2018); https://www.heraeus.com/media/media/hca/doc_hca/products_and_solutions_8/optics/Data_and_Properties_Optics_fused_silica_EN.pdf.

44. Heraeus Data Sheet Suprasil W (1970).

45. S. Menzel, S. Tappertzhofen, R. Waser, I. Valov, Switching kinetics of electrochemical metallization memory cells. *Phys. Chem. Chem. Phys.* **15**, 6945–6952 (2013).

46. O. Teschke, G. Ceotto, E. F. de Souza, Interfacial water dielectric-permittivity-profile measurements using atomic force microscopy. *Phys. Rev. E* **64**, 011605 (2001).

47. A. C. Torrezan, J. P. Strachan, G. Medeiros-Ribeiro, R. S. Williams, Sub-nanosecond switching of a tantalum oxide memristor. *Nanotechnology* **22**, 485203 (2011).
